# High murine blood persistence of phage T3 and suggested strategy for phage therapy

**DOI:** 10.1186/s13104-019-4597-1

**Published:** 2019-09-05

**Authors:** Philip Serwer, Elena T. Wright, John C. Lee

**Affiliations:** Department of Biochemistry and Structural Biology, The University of Texas Health Science Center, 7703 Floyd Curl Drive, San Antonio, TX 78229-3900 USA

**Keywords:** Average electrical surface charge density, Bacteremia, Innate immune systems, Pan-antibiotic-resistant bacteria, Rapid phage characterization, strategy for

## Abstract

**Objective:**

Our immediate objective is to determine whether infectivity of lytic podophage T3 has a relatively high persistence in the blood of a mouse, as suggested by previous data. Secondarily, we determine whether the T3 surface has changed during this mouse passage. The surface is characterized by native agarose gel electrophoresis (AGE). Beyond our current data, the long-term objective is optimization of phages chosen for therapy of all bacteremias and associated sepsis.

**Results:**

We find that the persistence of T3 in mouse blood is higher by over an order of magnitude than the previously reported persistence of (1) lysogenic phages lambda and P22, and (2) lytic phage T7, a T3 relative. We explain these differences via the lysogenic character of lambda and P22, and the physical properties of T7. For the future, we propose testing a new, AGE-based strategy for rapidly screening for high-persistence, lytic, environmental podophages that have phage therapy-promoting physical properties.

## Introduction

Bacterial infections become especially dangerous when caused by pan-antibiotic resistant bacteria [1–5]. A recourse is administration of phages, a process called phage therapy [6–12]. Phages kill the bacteria [6–12] and sometimes have positive immunomodulatory effects [8–11]. For bacteremias, the phages chosen are optimally lytic with persistence in blood of at least 3–5 h.

The persistence of studied phages, including lysogenic phages, P22 [13] and lambda [14], has missed this target. In murine blood, infectivity titer loss was, respectively, by over 6 orders and 1 order of magnitude in ~ 4 h. Lytic phage, T7, lost titer by over an order of magnitude in 1 h in an immunocompetent mouse, although not in an immunocompromised mouse [15]. These observations were negative indicators for phage therapy.

On the other hand, a discussion of mother mouse-to-fetus mouse migration of the T7-related phage, T3, includes the comment [16], “That phage remained viable in the animal (spleen) to 23 h was interesting.” This comment raises the possibility that phage T3 has a relatively high persistence in blood and, therefore, some phages for pathogenic hosts have similar high persistence. But, thus far, a direct test has not been performed for persistence in murine blood of phage T3. In the present study, we perform this test and find surprisingly high T3 persistence. We find evidence that the high T3 persistence is not associated with genetically selected T3 surface changes that occurred during the experiment. From these observations, we suggest future testing of a new strategy for more effective phage isolation for therapy of bacteremias.

## Main text

### Methods

#### Bacterial and phage strains

*Escherichia coli* BB/1 was the host for phage T3. T3 was received from Dr. F. W. Studier [17]. T3 is described and compared to its relative, T7, in Refs. [17–19].

*Escherichia coli* BB/1 was (1) propagated in 2xLB medium: 20 g tryptone, 10 g yeast extract, 5 g NaCl in 1.0 litre of Milli-Q filtered water (Millipore/Sigma) and (2) infected with phage T3 in 2xLB medium at 30 °C by use of procedures previously described [20]. Phages from a spontaneous lysate were purified by (1) precipitation with polyethylene glycol, (2) dilute DNase I treatment, (3) centrifugation through a cesium chloride step gradient, (4) buoyant density centrifugation in a cesium chloride density gradient and (5) dialysis against the following buffer: 0.15 M NaCl, 0.01 M Tris–Cl, pH 7.4, 0.001 M MgCl_2_. Details are in Ref. [18].

#### Inoculation of mice

Mice were inoculated in the laboratory (~ 10:00 A.M.) via IP injection (possible also for phage therapy) with 100 μl of 4.0 × 10^12^ phage T3 per ml in the final buffer of purification (previous paragraph). Blood samples were drawn through the tail vein at times indicated. No anesthesia or analgesia was used.

The mice were wild-type, young (10–12 weeks old), ~ 20 g, C57BL/6 females, disease-free and healthy, purchased from Harlan and maintained with 12 h of light/12 h of dark cycles at 22 °C in sterile rooms and sterile cages at the authors’ institution. Standard irradiated rodent chow (Harlan Teklad-485) and water were provided ad libitum. Each cage had (1) no more than five mice and (2) a stainless-steel wire bar lid and water bottle. Facility access was limited to trained investigators and staff wearing protective clothing: head wares, face masks, gown, gloves and foot wares. The university provided state-of-the-art husbandry, veterinary care and management support. After phage infection and blood sampling, the investigators observed mice for signs of adverse effects. Mice were handled and euthanized according to the protocol (Number 170074x) approved by the Institutional Animal Care and Use Committee of the University of Texas Health Center at San Antonio, TX, USA. This institution has an Animal Welfare Assurance on file with the NIH Office of Laboratory Animal Welfare: Assurance Number, A3345-01. Animal care and use was in accordance with the NRC Publication, as revised in 2011, “Guide for the Care and Use of Laboratory Animals,” and other applicable federal regulations.

#### Infectivity assay

Titers of infective phages were obtained by use of standard plaque-forming procedures [21] with a 0.7% top layer agar gel in 2xLB medium, except as indicated below. Dilutions for plaque count were made in 0.5 M NaCl, 0.01 M Tris–Cl, pH 7.4, 0.001 M MgCl_2_, 1.0 mg/ml gelatin. Plates were incubated at 37 °C; dilutions were adjusted so that titers were based on at least 200 plaques, sampling error less than 7%.

#### Native agarose gel electrophoresis (AGE)

AGE of plaque-associated phage particles was performed after formation of plaques in an upper layer gel cast from the following molten mixture of two low-melt agarose preparations (Lonza): 0.5% SeaPlaque + 0.5% SeaPrep. To release phages from a plaque supported by this gel, ~ 10 μl of gel was incubated at 39 °C for 1.0 h in a narrow glass tube (inner diameter = 4.5 mm), with vortexing every 15 min. Then, DNA was digested by adding 1.1 μl DNase I (1.0 mg/ml) in 0.045 M MgCl_2_ and incubating for 1.0 h at 30 °C. Before AGE, 8.9 μl of the following was added: tracking dye (200 μg/ml bromophenol blue) and neutral, density-increasing compound (11.2% sucrose) in 0.09 M Tris–acetate, pH 8.3. Procedure was adapted from Ref. [22].

AGE was performed in a 1.0% horizontal, submerged agarose (Seakem LE; Lonza) gel for 18.0 h, at 1.0 V/cm, in electrophoresis buffer: 0.09 M Tris–acetate, pH 8.3, 0.001 M MgCl_2_. Electrophoresis (1) was started 1.0 h. after the loading of samples, this delay having been introduced to reduce buffer discontinuities, and (2) was accompanied by buffer circulation (> 100 ml/min) through a temperature-controlled water bath, beginning at 1.0 h. after the start of electrophoresis. The gel temperature was 25 ± 0.5 °C. After electrophoresis, particles were visualized by staining with GelStar (1:25,000 dilution of GelStar solution from Lonza; nucleic acid-specific) and ultraviolet light illumination.

### Results

#### Lifetime of phage T3 in mouse blood

**Fig. 1:**
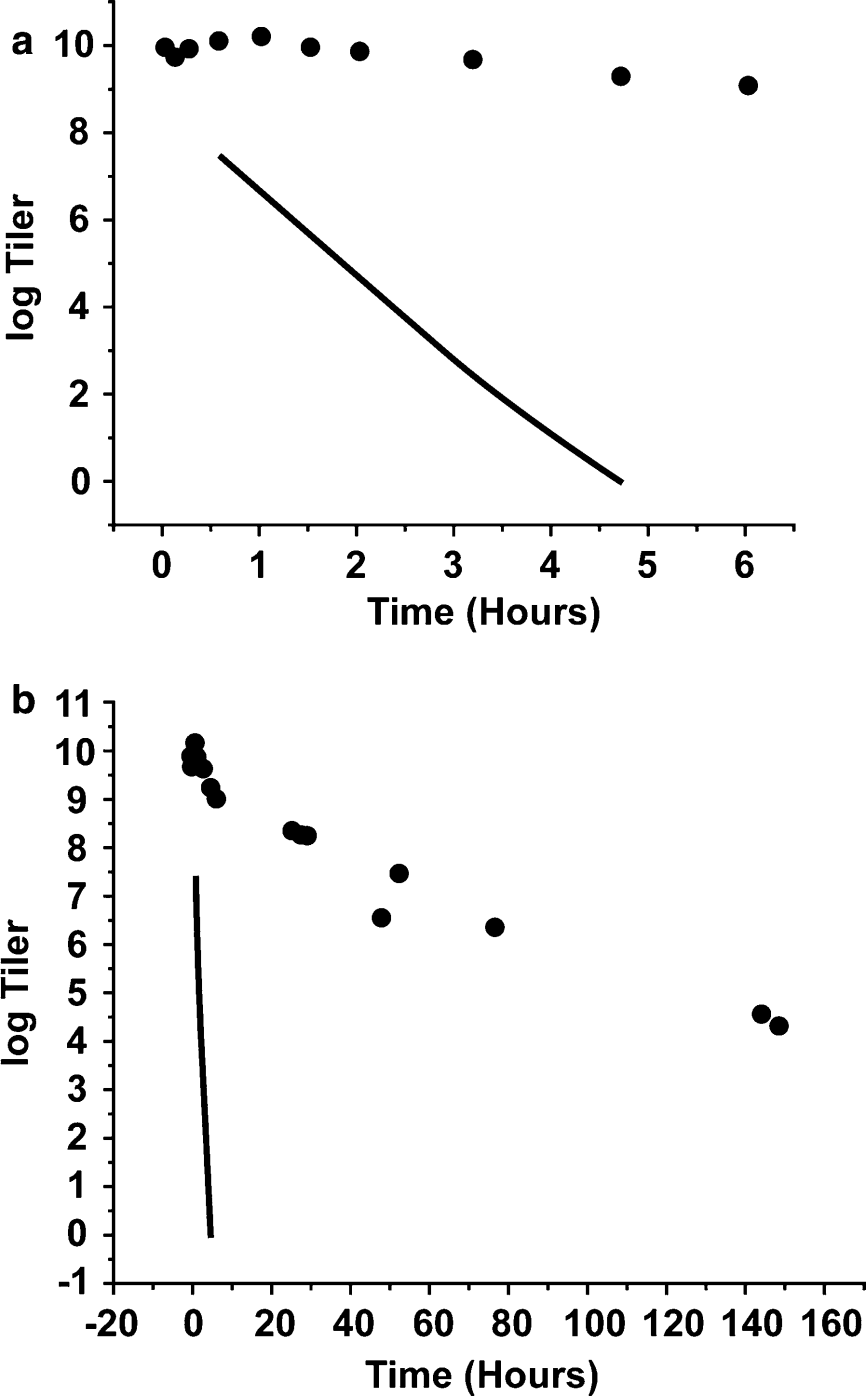
The infectivity titer of phage T3 in mouse blood vs. time. **a** Early times. **b** All times. Solid line, P22 [13]. Filled circles, T3

After IP injection of phage T3, the T3 blood titer did not significantly decrease for the first 3–4 h (filled circles in Fig. 1a). Subsequent decrease in titer occurred and continued for 8 days (Fig. 1b). Titer decreased by about an order of magnitude per day.

To illustrate the dramatic contrast with the early time data for phage P22, the data for P22 from Ref. [13] are plotted as a solid line in Fig. 1a. The contrast continued at the later times (Fig. 1b). The standard error of titers was less than 7%.

#### Native gel electrophoresis

We did the following to rapidly determine whether T3 had experienced mouse passage-induced selection sufficient to cause inherited changes in its surface. We performed AGE of phages that had been propagating in plaques previously generated by infectivity assays of blood samples taken at several different times.

**Fig. 2:**
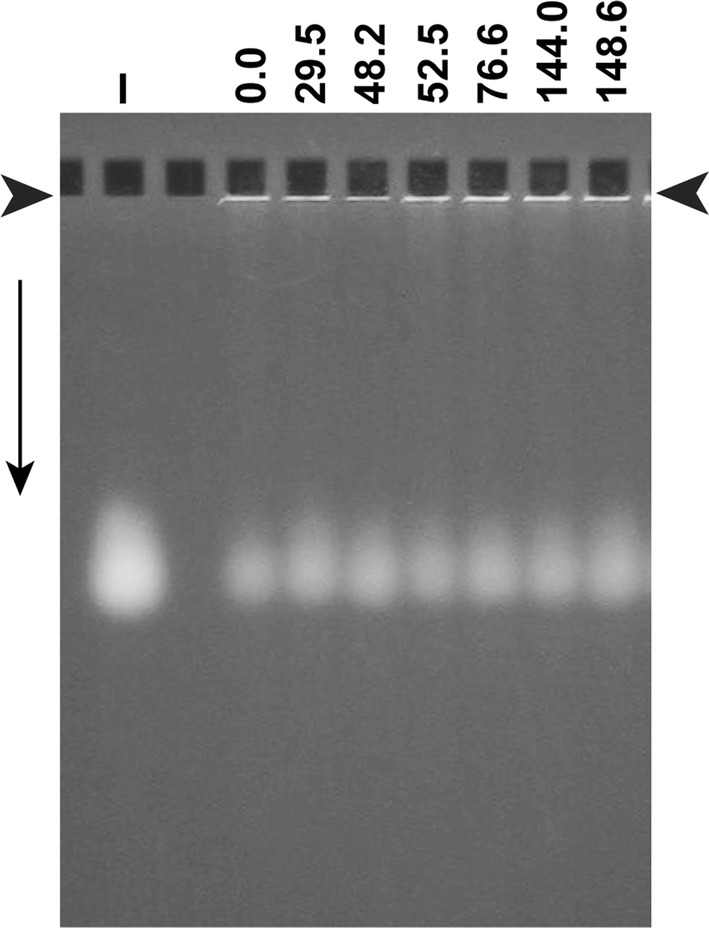
AGE of T3 phage from plaques obtained at the time (h), post-inoculation, indicated above a lane. The plaques were from the experiment in Fig. 1

The AGE produced a single band at a position that did not did not vary with time after mouse-injection (Fig. 2; time [h] is above a lane); this position was the same as it was for the inoculum (I lane). Migration speed during AGE decreases with increase in particle dimensions (radius, in this case) and increases linearly with the magnitude of the average electrical surface charge density, σ [23–25]; σ is negative. If either radius or σ had varied by 3% or more, change in band position would have been seen [25].

### Discussion

#### High T3 persistence in blood: a possible explanation

The following characteristics of phage T3 are possible causes of high persistence. First, T3 is a lytic phage. As such, all ancestral T3 DNAs experienced evolutionary pressure while packaged in a phage particle that was extracellular [26, 27]. This implies selection for high persistence if the extracellular environment included animals/humans. In contrast, ancestral lysogenic phage DNAs sometimes were not so selected [26, 27].

Second, phage T3 was almost certainly isolated from feces or sewage [28]. This origin implies passages through humans and/or animals. Thus, T3 was, indeed, likely to have been selected for high persistence. The data of Fig. 2 suggest that the T3 surface was not further modified by selection during the experiment of Fig. 1.

Finally, one suspects that the podovirus aspect of T3 [19] is a factor. Podoviruses have relatively short tails and tail fibers [29]. Thus, entanglement-driven loss is expected to be less than it is for longer-tailed phages, i.e., siphoviruses and myoviruses. The T3 relative, T7, also has these advantages.

#### T3 vs. T7

In comparison to T3, T7 has three persistence-limiting physical properties that possibly explain the observation of relatively low T7 murine persistence in Ref. [15]. (1) T7, but not T3, has an AGE-detected, non-infective state (second AGE band) into and out of which it can be environmentally induced. Adoption of this non-infective state could cause either the appearance or the reality, of low persistence [30]. (2) T7, but not T3, is adherent to agarose gels during AGE, suggesting that T7 is generally “stickier” than T3 [18, 31]. This could cause low persistence via adsorption of T7 to epithelia. Possibly linked to the lower-than-T7 stickiness of T3 is the relatively high absolute value of the negative T3 σ, 1.28 × the negative σ of T7 [18]. (3) T7 phage is less elevated temperature-stable than T3 (unpublished observations), possibly because of a genome that is more tightly packed [18].

Single-plaque AGE (Fig. 2) screens directly for properties (1) and (2). The following data suggest that single-plaque AGE can also screen for high persistence via screening for negative σ high in magnitude. Experience with non-phage drug delivery vehicles has been that overall persistence is increased by negative σ and decreased by positive σ [32, 33], although some innate immune systems do remove particles with negative σ [34].

#### Proposed improvements in isolating phages for phage therapy

The finding made here of a high-persistence phage suggests that, beyond the current practice of selecting clear plaque-forming phages [35], in the future, bacteremia-targeted phages should be screened for high persistence. But, repeating the experiment of Fig. 1 is time-consuming enough to look for a proxy screen. The discussion in the previous section suggests testing the possibility that the following is an indicator of high phage persistence: a single AGE band associated with negative σ relatively high in magnitude. Values of σ are rapidly obtained by single-plaque AGE (Fig. 2) through two or more agarose gels of different concentration [36]. This can be done during the initial single-plaque purification.

A rapid, simple, partial screen for podophages is also achievable during single-plaque purification. This is done by comparing the diameter of plaques in a relatively low-concentration (e.g., 0.3%) agarose supporting gel with the diameter in a higher concentration (e.g., 1.2%) agarose supporting gel (example [37]). Podophages will be preferentially identified via low dependence of plaque diameter on supporting gel concentration, because podophages are typically smaller than other DNA phages. Podophage classification was established for phages that had previously been found [38] most effective for the phage therapy of murine *E. coli*-bacteremias and meningitis [39]. More extensive data appear not to be available.

For improving phage therapy of bacteremias, factors other than those discussed above can be involved. But, future testing of the correlation of podophage σ with phage therapy effectiveness is a reasonable beginning.

## Limitations

1. We have not determined, in detail, how well σ, lytic character and podovirus character correlate with the persistence of phages other than T3 and T7.
2. We have not tested how reliably our recommended procedure isolates podophages.
3. None of the recommended procedures have been tested for therapy.

## Data Availability

All data generated or analyzed during this study are included in this published article.

## Abbreviations

AGE: native agarose gel electrophoresis
σ: average electrical surface charge density

## References

[1] Venter H, Henningsen ML, Begg SL (2017). Antimicrobial resistance in healthcare, agriculture and the environment: the biochemistry behind the headlines. Essays Biochem.

[2] Martens E, Demain AL (2017). The antibiotic resistance crisis, with a focus on the United States. J Antibiotechnol.

[3] Zaman SB, Hussain MA, Nye R, Mehta V, Mamun KT, Hossain NA (2017). Review on antibiotic resistance: alarm bells are ringing. Cureus..

[4] Aslam B, Wang W, Arshad MI, Khurshid M, Muzammil S, Rasool MH, Nisar MA, Alvi RF, Aslam MA, Qamar MU, Salamat MKF, Baloch Z (2018). Antibiotic resistance: a rundown of a global crisis. Infect Drug Resist..

[5] Bloom DE, Black S, Salisbury D, Rappuoli R (2018). Antimicrobial resistance and the role of vaccines. Proc Natl Acad Sci USA.

[6] Moelling K, Broecker F, Willy CA (2018). Wake-up call: we need phage therapy now. Viruses..

[7] Roach DR, Debarbieux L (2017). Phage therapy: awakening a sleeping giant. Emerg Top Life Sci..

[8] Górski A, Jończyk-Matysiak E, Łusiak-Szelachowska M, Międzybrodzki R, Weber-Dąbrowska B, Borysowski J (2017). The potential of phage therapy in sepsis. Front Immunol..

[9] Hodyra-Stefaniak K, Miernikiewicz P, Drapała J, Drab M, Jończyk-Matysiak E, Lecion D, Kaźmierczak Z, Beta W, Majewska J, Harhala M, Bubak K, Kłopot A, Górski A, Dąbrowska K (2015). Mammalian host-versus-phage immune response determines phage fate *in vivo*. Sci Rep..

[10] Krut O, Bekeredjian-Ding I (2018). Contribution of the immune response to phage therapy. J Immunol..

[11] Roach DR, Leung CY, Henry M, Morello E, Singh D, Di Santo JP, Weitz JS, Debarbieux L (2017). Synergy between the host immune system and bacteriophage is essential for successful phage therapy against an acute respiratory pathogen. Cell Host Microbe.

[12] Speck Peter, Smithyman Anthony (2015). Safety and efficacy of phage therapy via the intravenous route. FEMS Microbiology Letters.

[13] Merril CR, Scholl D, Adhya SL (2003). The prospect for bacteriophage therapy in Western medicine. Nat Rev Drug Discov..

[14] Merril CR, Biswas B, Carlton R, Jensen NC, Creed GJ, Zullo S, Adhya S (1996). Long-circulating bacteriophage as antibacterial agents. Proc Natl Acad Sci USA.

[15] Srivastava AS, Kaido T, Carrier E (2004). Immunological factors that affect the in vivo fate of T7 phage in the mouse. J Virol Meth..

[16] Kulangara AC, Sellers MI (1959). Passage of bacteriophages from mother to foetus in the rat. Proc Soc Exp Biol Med.

[17] Studier FW (1979). Relationships among different strains of T7 and among T7-related bacteriophages. Virology.

[18] Serwer P, Watson RH, Hayes SJ, Allen JL (1983). Comparison of the physical properties and assembly pathways of the related bacteriophages T7, T3 and phi II. J Mol Biol.

[19] Pajunen MI, Elizondo MR, Skurnik M, Kieleczawa J, Molineux IJ (2002). Complete nucleotide sequence and likely recombinatorial origin of bacteriophage T3. J Mol Biol.

[20] Serwer P, Wright ET, Liu Z, Jiang W (2014). Length quantization of DNA partially expelled from heads of a bacteriophage T3 mutant. Virology.

[21] Adams MH (1959). Bacteriophages.

[22] Serwer P, Hayes SJ, Watson RH, Khan SA (1995). Gel electrophoretic analysis of bacteriophage assembly intermediates in bacteriophage plaques. Appl Theor Electrophor.

[23] Shaw DJ (1969). Electrophoresis.

[24] Stellwagen NC (2009). Electrophoresis of DNA in agarose gels, polyacrylamide gels and in free solution. Electrophoresis.

[25] Casjens S, Wyckoff E, Hayden M, Sampson L, Eppler K, Randall S, Moreno ET, Serwer P (1992). Bacteriophage P22 portal protein is part of the gauge that regulates packing density of intravirion DNA. J Mol Biol.

[26] Clokie MR, Millard AD, Letarov AV, Heaphy S (2011). Phages in nature. Bacteriophage..

[27] Rohde C, Wittmann J, Kutter E (2018). Bacteriophages: a therapy concept against multi-drug-resistant bacteria. Surg Infect (Larchmt).

[28] Abedon ST (2000). The murky origin of Snow White and her T-even dwarfs. Genetics.

[29] Fokine A, Rossmann MG (2014). Molecular architecture of tailed double-stranded DNA phages. Bacteriophage..

[30] Gabashvili IS, Khan SA, Hayes SJ, Serwer P (1997). Polymorphism of bacteriophage T7. J Mol Biol.

[31] Serwer P, Hayes SJ (1982). Agarose gel electrophoresis of bacteriophages and related particles. I. Avoidance of binding to the gel and recognizing of particles with packaged DNA. Electrophoresis..

[32] Lim C, Sim T, Hoang NH, Jung CE, Lee ES, Youn YS, Oh KT (2017). A charge-reversible nanocarrier using PEG-PLL (-*g*-Ce6, DMA)-PLA for photodynamic therapy. Int J Nanomedicine..

[33] Xu C, Song RJ, Lu P, Chen JC, Zhou YQ, Shen G, Jiang MJ, Zhang W (2018). pH-triggered charge- reversal and redox-sensitive drug-release polymer micelles codeliver doxorubicin and triptolide for prostate tumor therapy. Int J Nanomed.

[34] Fröhlich E (2012). The role of surface charge in cellular uptake and cytotoxicity of medical nanoparticles. Int J Nanomed.

[35] Hyman P (2019). Phages for phage therapy: isolation, characterization, and host range breadth. Pharmaceuticals (Basel)..

[36] Serwer P, Khan SA, Griess GA (1995). Non-denaturing gel electrophoresis of biological nanoparticles: viruses. J Chromatogr A.

[37] Serwer P, Hayes SJ, Zaman S, Lieman K, Rolando M, Hardies SC (2004). Improved isolation of undersampled bacteriophages: finding of distant terminase genes. Virology.

[38] Smith HW, Huggins MB (1982). Successful treatment of experimental *Escherichia coli* infections in mice using phage: its general superiority over antibiotics. J Gen Microbiol.

[39] Baig A, Colom J, Barrow P, Schouler C, Moodley A, Lavigne R, Atterbury R (2017). Biology and genomics of an historic therapeutic *Escherichia coli* bacteriophage collection. Front Microbiol..

